# New-Onset Multiple Sclerosis in Pregnancy: Diagnostic Approaches and Treatment Dilemmas

**DOI:** 10.7759/cureus.74685

**Published:** 2024-11-28

**Authors:** Nathallie George, Cledervern Brebnor Des Isles, Ghazal Tannous

**Affiliations:** 1 Obstetrics and Gynecology, Nottingham University Hospitals NHS Trust, Nottingham, GBR; 2 Obstetrics and Gynecology, United Lincolnshire Hospitals NHS Trust, Lincoln, GBR

**Keywords:** maternal mortality or death, multiple sclerosis, neurological autoimmune disorders, preconception counseling, pregnancy

## Abstract

Multiple sclerosis (MS) is an autoimmune disease of the CNS affecting the brain, spinal cord, and optic nerves. Research consistently shows that relapse rates in MS decrease during pregnancy, particularly in the third trimester. However, these rates increase postpartum, especially within the first three months after delivery, returning to prepregnancy levels. Importantly, studies indicate that pregnancy does not alter the overall course or progression of MS. While there are detailed accounts of new-onset MS presentations, documentation of cases that first present during pregnancy remains scarce. This case highlights a unique presentation that challenges the current understanding, as it deviates from previously reported literature. We emphasize the distinctive MRI findings that were crucial for diagnosing MS and discuss the difficulties in differentiating it from other neurological conditions. The case also underscores the importance of individualized care and a multidisciplinary approach, including preconception counseling, to reduce relapse risk and long-term disability while minimizing potential harm to both the patient and the offspring. We report the case of a 29-year-old gravida 2, para 1 female with an uneventful antenatal course until 25 weeks of gestation, when she developed new-onset neurological symptoms, beginning with numbness and weakness in her limbs. Initially, she experienced pins and needles in her left hand, which rapidly progressed to reduced sensation, immobility, and urinary retention. By 29 weeks, her symptoms had worsened, resulting in near-paralysis of both legs and the need for a prolonged indwelling catheter. At 35 weeks, she was referred to a tertiary care center. MRI scans revealed multiple hyperintense lesions in both the brain and spinal cord, particularly in the centrum semiovale and the C3-C4 regions, indicative of demyelination. Neuromyelitis optica spectrum disorder and anti-MOG antibody testing were negative, while CSF analysis revealed oligoclonal bands. Based on these findings, she was diagnosed with MS rather than transverse myelitis or clinically isolated syndrome. The patient underwent immunotherapy, including intravenous methylprednisolone and plasmapheresis, which resulted in significant improvement in leg mobility and sensory deficits. By six weeks postpartum, she demonstrated functional recovery, although some symptoms, such as sensory deficits and an abnormal gait, persisted. This case illustrates the diagnostic challenges of distinguishing MS from other neurological conditions during pregnancy. Key findings included hemiplegia, sensory loss, multiple MRI lesions in both the brain and spinal cord, and the presence of oligoclonal bands in CSF. The progressive symptomatology during pregnancy, alongside these clinical features, can guide clinicians in recognizing and managing similar cases.

## Introduction

The most recent Mothers and Babies: Reducing Risk through Audit and Confidential Enquiries (MBBRACE-UK) report indicated that, following cardiac causes, neurological diseases remain the second most common cause of indirect maternal deaths in the United Kingdom, a trend that has persisted over the past 20 years [1]. While sensory abnormalities are common during pregnancy and typically resolve after delivery, this report presents a rare and challenging case of new-onset multiple sclerosis (MS) during pregnancy, highlighting the diagnostic and therapeutic dilemmas faced by clinicians. Notably, our patient first presented with sensory deficits in the late second trimester. Typically, patients are reassured that sensory abnormalities during pregnancy will resolve postpartum. However, this expectation requires clinicians to have an in-depth understanding of the physiological changes affecting the nervous system during pregnancy, as well as the ability to diagnose and manage neurological conditions that may arise during this time [2].

MS is an autoimmune disease of the CNS, characterized by demyelination in the brain, spinal cord, and optic nerves [3]. Symptoms such as muscle weakness, vision problems, and cognitive difficulties can significantly impact quality of life. Kantarici described various stages of MS, ranging from the initial diagnosis to advanced stages, encompassing both early and late stages within the disease spectrum [4]. MS typically manifests in one of three forms: relapsing-remitting MS (RRMS), primary progressive MS (PPMS), or secondary progressive MS (SPMS), each with distinct patterns of symptom progression. The interaction between MS and pregnancy is complex. Pregnancy, especially in the third trimester, is known to reduce relapse rates due to hormonal changes, but relapse rates tend to increase postpartum. While MS does not generally affect fertility or pregnancy outcomes, its onset during pregnancy is exceedingly rare, presenting diagnostic and therapeutic challenges. This report discusses a unique case of new-onset MS during pregnancy, emphasizing the difficulties in distinguishing it from conditions such as transverse myelitis (TM) and neuromyelitis optica spectrum disorder (NMOSD). It underscores the importance of a multidisciplinary approach to diagnosis and management, aiming to minimize risks to both the mother and the child.

Before being diagnosed with MS, patients are often categorized into one of two syndromes. The first, known as clinically isolated syndrome (CIS), refers to a single episode of CNS demyelination that does not meet the diagnostic criteria for MS [5]. The second is radiologically isolated syndrome, where MS-related findings are incidentally discovered on an MRI scan, but the patient remains asymptomatic and has no alternative explanation for the findings [6]. Literature suggests that 30-70% of patients who develop CIS are later diagnosed with MS [7].

The signs and symptoms of MS are highly variable, making it challenging to ascertain the specific type of disease. Each form of MS has its own distinctive features, although some overlap can occur [8]. Some patients present with CIS, while others may present in the progressive or relapsing phase of the disease. RRMS is diagnosed in approximately 80-85% of cases and is characterized by episodes of relapse followed by complete or partial remission until another relapse occurs [9,10]. PPMS, found in 10-20% of cases, is marked by a gradual and continuous decline in neurological function without periods of remission or improvement [9]. SPMS, commonly diagnosed within 10 years after an initial RRMS diagnosis, occurs in approximately 50% of those with RRMS [11]. This form of MS is characterized by a steady worsening of neurological function, independent of relapses [12]. In SPMS, patients may experience an “active phase” initially with relapses, which do not fully remit. Without treatment, many will progress to a “non-active” phase, where there is continued disability without relapses [12].

The introduction of long-term disease-modifying therapies (DMTs) has changed the management of MS, reducing the number of patients who progress to SPMS. These therapies are often used to mitigate neurological symptoms, particularly in those presenting with CIS, and can delay an eventual MS diagnosis [13,14]. It is also noteworthy that 20-30% of MS patients do not experience significant disability even 20 years after diagnosis [15].

There is ongoing debate in the literature regarding whether pregnancy can delay the first episode of demyelination or CIS. Despite extensive research, further clarification is needed regarding the effect of MS on pregnancy [16]. However, it is well established that relapse rates are significantly reduced in pregnant women with mild MS. Pregnancy is believed to have a protective effect on MS relapses, although relapse rates do not decrease in women treated with high-efficacy therapies [17]. It has also been suggested that pregnancy after a diagnosis of MS does not increase the risk of long-term disability [18]. However, it remains unclear whether this is also true for women diagnosed during pregnancy, as seen in our case [19].

The literature reveals a lack of consensus regarding the effect of pregnancy on the onset of MS. Some studies support the notion that pregnancy provides a protective effect against relapses, while others suggest that this benefit may be lost in women with active disease receiving high-efficacy treatments [16,20]. Confavreux et al. [21] conducted the first large prospective observational study, the Pregnancy in Multiple Sclerosis (PRIMS) study, which analyzed 269 pregnancies. This study demonstrated a reduction in relapse rates during pregnancy, particularly in the third trimester, with an increase in relapses during the first three months postpartum, returning to prepregnancy levels thereafter.

A contentious issue discussed in the literature is whether pregnancy can delay the first episode of demyelination or the confirmation of an MS diagnosis [22]. While MS does not typically affect fertility or pregnancy outcomes, its onset during pregnancy is exceedingly rare and poses diagnostic and therapeutic challenges. This report discusses a unique case of new-onset MS during pregnancy, highlighting the challenges in differentiating it from conditions such as TM and NMOSD. It underscores the importance of a multidisciplinary approach to diagnosis and management, aiming to minimize risks to both the mother and the child.

## Case presentation

A 29-year-old gravida 2, para 1 woman presented at 25 weeks gestation with new-onset neurological symptoms, beginning with numbness and weakness in her limbs. Initial symptoms included pins and needles in her left hand, which rapidly progressed to reduced sensation, immobility, and urinary retention. A neurological examination revealed intact cranial nerves and orientation to time, place, and person. There was reduced power (2/5) in both the left and right lower limbs across all directions for hip flexion and extension, knee flexion and extension, and dorsiflexion and extension. Power in the upper limbs was 5/5 bilaterally. Sensation was decreased in the left lower limb, but intact in the right lower and upper limbs. Reduced anal tone was also noted, while all neurological reflexes, including deep tendon reflexes, were within normal limits. By 29 weeks, her symptoms worsened, leading to near-paralysis of both legs and the need for a prolonged indwelling catheter. She also developed a right leg deep vein thrombosis at 32 weeks gestation due to immobility.

Prior to the onset of symptoms, she had an uneventful antenatal course and was independent, working as a carer in a care home. Her medical history included depression and anxiety, managed with sertraline and perinatal mental health input. She had no history of smoking or alcohol consumption and no family history of MS or other neurological conditions. A multidisciplinary team was consulted for her management, including an obstetrician, anesthetist, neurologist, and pediatrician. The first MRI of the brain and spinal cord led to an initial diagnosis of TM after review by the neurology team. The brain MRI showed two small demyelinating lesions in the subcortical area, while the spinal MRI revealed six short-segment and larger cord lesions affecting the cervical, thoracic, and distal thoracic regions, as well as the conus medullaris. The mid-thoracic lesion was slightly extensible, but only two vertebral lengths. TM was initially diagnosed, as she was almost paraplegic, bedbound, and had sphincter dysfunction. She was then transferred to a tertiary center for specialist care. Acute immunotherapy was administered in the form of intravenous methylprednisolone, followed by oral steroids and plasmapheresis (plasma exchange) by the renal team. Notably, she showed significant improvement in leg movement following plasmapheresis.

**Figure 1:**
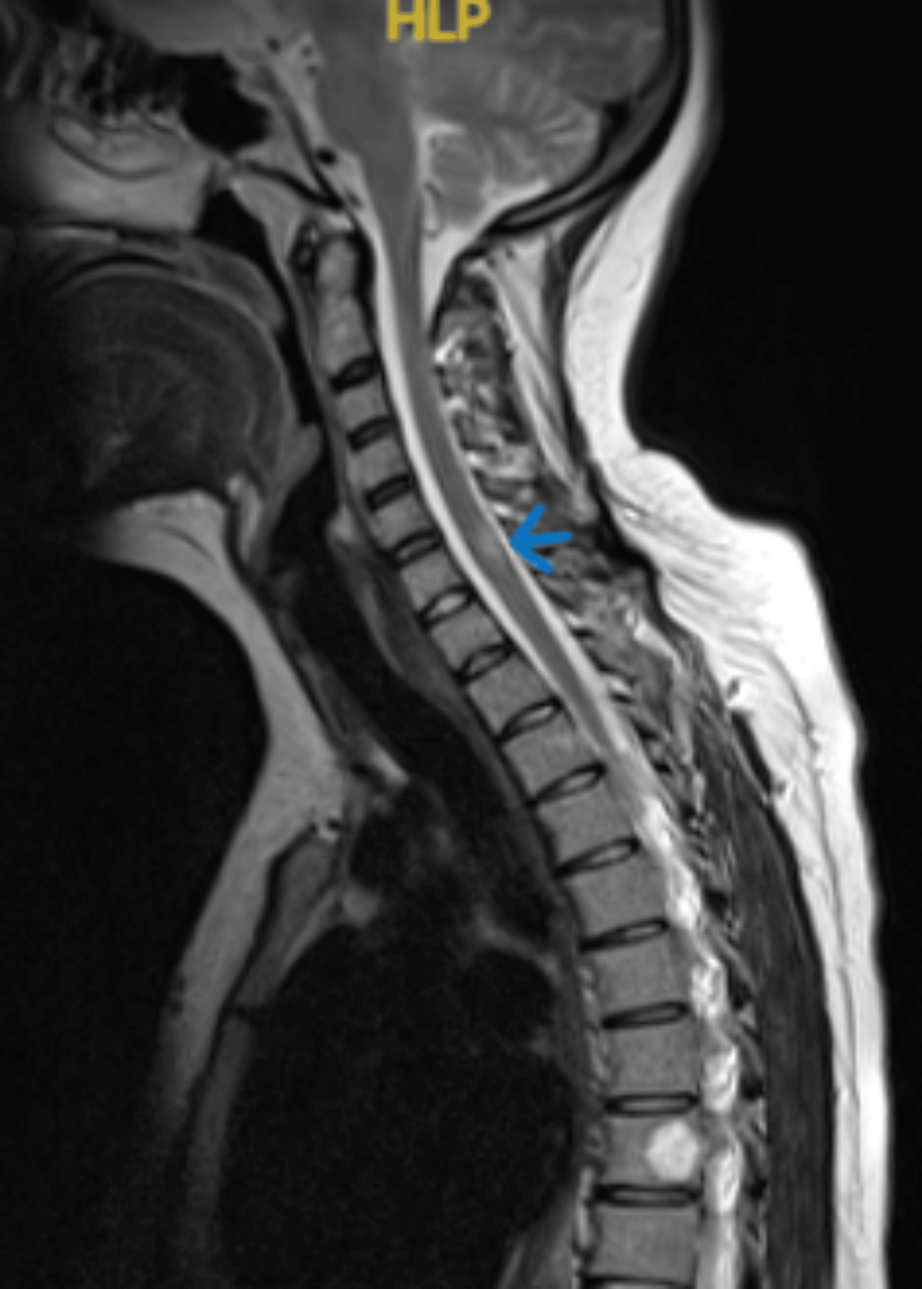
T2-weighted MRI of the cervical spine showing a hyperintense lesion at the C3-C4 level (indicated by the blue arrow), suggestive of demyelination

**Figure 2:**
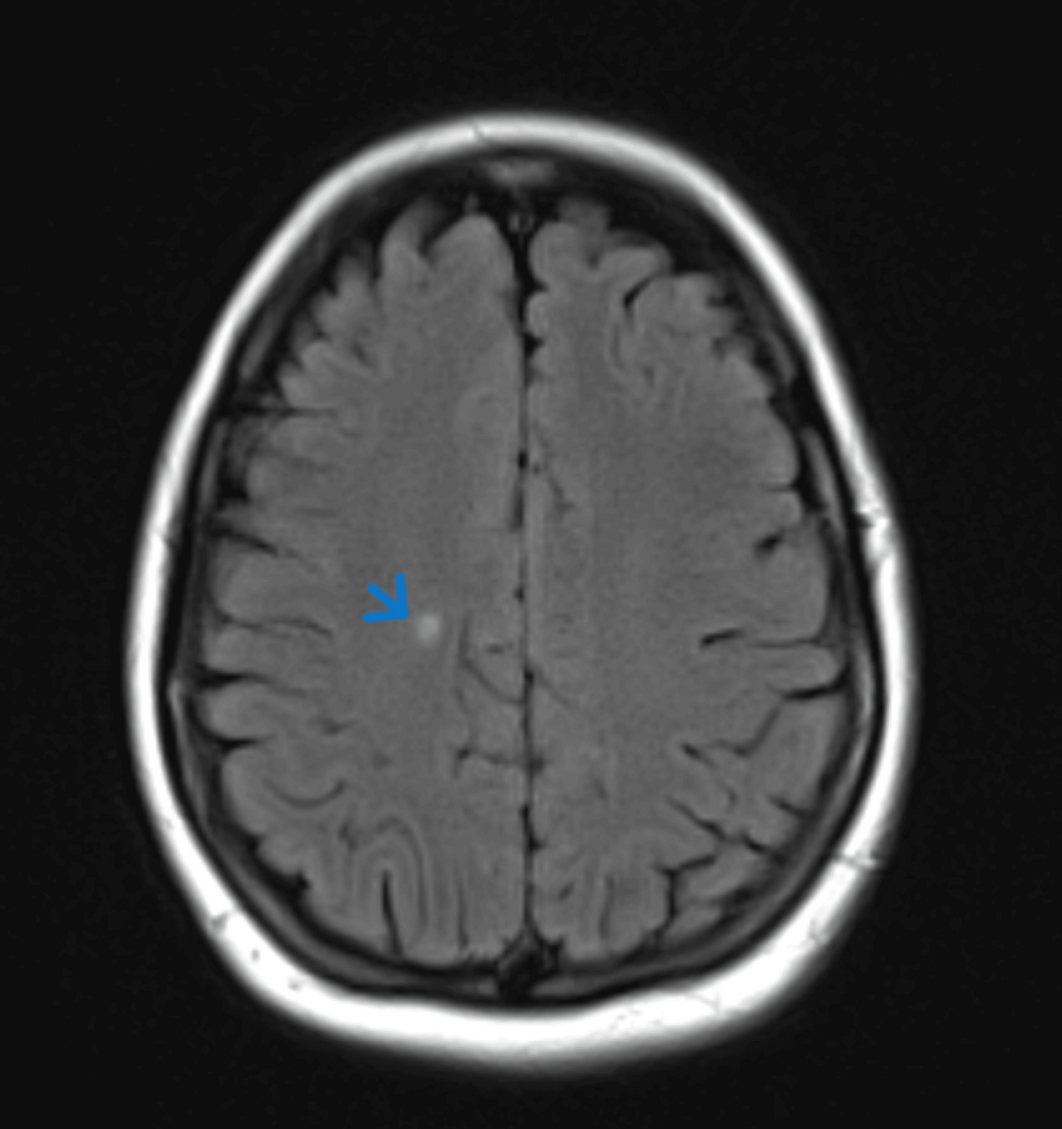
T2-weighted MRI of the brain showing a hyperintense focal lesion in the right centrum semiovale (indicated by the blue arrow), consistent with demyelination

Further diagnostic evaluation was carried out, including an MRI of the brain, lumbar, and sacral spine. There was no evidence of significant degenerative changes in the lumbar spine or bulging lumbar intervertebral discs that could indicate neural compression or acute cauda equina compression. However, faint T2/STIR hyperintense signal foci were noted in the conus medullaris, as well as in the thoracic and cervical spinal cord at the C2, C3-4, C6, T3, T11, and T12 vertebral discs (Figure 1). These lesions correlated with the patient's symptoms of lower limb weakness and urinary retention, supporting a diagnosis of demyelination consistent with MS. The rest of the spinal cord appeared unremarkable, except for a few focal lesions that were hyperintense on T1- and T2-weighted images, corresponding to typical hemangiomas at the T7 and T9 vertebral bodies. A contrast-enhanced MRI of the cervicothoracic spine was recommended for further evaluation to exclude underlying spinal cord pathology. Additionally, a T2-weighted MRI of the brain and spine showed hyperintense focal lesions in the right centrum semiovale (Figure 2), left parieto-occipital subcortical white matter, and cervicothoracic spinal cord, likely representing demyelination plaques of MS. These lesions demonstrate dissemination in space as required by the McDonald criteria for MS diagnosis.

Her anti-aquaporin-4 antibody (AQP4) and anti-myelin oligodendrocyte glycoprotein (anti-MOG) tests were both negative. A full autoimmune screen, including tests for antinuclear antibodies, antineutrophil cytoplasmic antibodies, extractable nuclear antigens, and cardiolipin, was also negative. Serology testing for infections, including hepatitis B, hepatitis C, HIV, and syphilis, returned negative results. CSF analysis following lumbar puncture revealed 12 white cells (70% lymphocytes and 30% polymorphs), 14 red cells, a protein level of 323 mg/dL, and glucose of 3.6 mmol/L. Serum glucose and viral PCRs were negative, but oligoclonal bands were present.

An intrapartum care plan was established following neurological and anesthetic pre-assessment. The delivery options, including vaginal delivery and cesarean section, were discussed. The patient opted for an elective cesarean section at 38 weeks, following recurrent episodes of reduced fetal movements and concerns about a small gestational-age baby. Measures were taken to minimize the risk of neurological injury and ensure support during delivery. Postoperatively, her recovery was uneventful.

She gradually regained mobility, initially requiring daily physiotherapy and carer assistance for several weeks. Six weeks postpartum, she was able to walk, although still using a Zimmer frame for distances of 50-100 meters without needing to rest. She continued to have difficulty initiating micturition.

By four months postpartum, she could stand and walk with the assistance of a frame, occasionally using a stick. Indoors, she could walk unaided, although her gait remained abnormal. She was fitted with bilateral ankle-foot orthoses for foot drop, which improved with physiotherapy. While her leg strength improved, sensory deficits and pain persisted. At a follow-up neurological review, her Expanded Disability Status Scale score ranged from 4.5 to 6. At this stage, she could walk 300 meters without aid or rest. Her bladder function returned to normal, and she no longer required an indwelling catheter.

The patient continues to follow up with the neurology and physiotherapy teams. Six months postpartum, she reported persistent aching in her hands, which sometimes caused fatigue but did not impair her overall function. She also described discomfort in her back and legs. She was offered DMT to reduce relapse and long-term disability but declined to start treatment, citing her desire to breastfeed for one year.

## Discussion

The de novo presentation and sudden deterioration of the patient’s condition made an initial diagnosis challenging. Conditions such as TM, NMOSD, and MS were all considered. After reviewing the evidence, it was concluded that the patient had MS rather than TM or CIS. In this report, we review each condition, provide support for our diagnosis, and highlight the distinctive MRI features that aid in making the correct diagnosis in such complex cases.

Initially, TM was considered due to the patient’s loss of sensory function and bladder dysfunction. TM is a rare inflammatory disorder of the spinal cord that damages the myelin, affecting the sensory, motor, and autonomic systems [23]. It is diagnosed when there is a rapid onset of neurological symptoms or subacute involvement of one or more spinal cord segments in patients without compressive cord lesions [24]. Half of TM patients show moderate lymphocytosis in CSF analysis [25].

Next, we considered NMOSD, a rare autoimmune disease affecting the spinal cord and optic nerve [26]. A few years ago, NMOSD was classified as a variant of MS, but the discovery of antibodies against AQP4-IgG led to its classification as a distinct disease [27]. In more than 75% of cases, AQP4-IgG antibodies are present [27]. We also considered the possibility of MOG antibody disease, a recently identified autoimmune disorder with a preference for optic nerve and spinal cord involvement [28]. This condition was ruled out as the patient tested negative for both AQP4-IgG and MOG-IgG, helping to exclude NMOSD and MOG-associated disease. Seronegative NMOSD, which includes patients who meet NMOSD criteria without the presence of either AQP4-IgG or MOG-IgG, was considered but ultimately ruled out based on these negative results [29].

MRI plays a crucial role in diagnosing NMOSD, particularly in AQP4-negative cases. Typical MRI findings in NMOSD include bilateral, longitudinally extensive optic neuritis, usually affecting more than half of the optic nerve and extending to the chiasm and optic tract [30]. In contrast, MOG antibody-related optic neuritis is typically localized to the anterior part of the optic nerve and exhibits more pronounced inflammation compared to AQP4-IgG-positive cases [31]. MOG antibody disease also has a distinct predilection for the conus medullaris [32].

The diagnosis of MS requires consideration of clinical symptoms, imaging findings, and laboratory results and is based on the McDonald diagnostic criteria, which have been updated several times over the past 15 years [33]. The 2024 revisions to the McDonald criteria, announced at the 40th Congress of the European Committee for Treatment and Research in Multiple Sclerosis (ECTRIMS), emphasize the need for evidence of typical damage in the brain or spinal cord, demonstrated by MRI lesions. These revisions include the optic nerve as a new location for demonstrating dissemination in space, in addition to infratentorial, periventricular, cortical/juxtacortical, and spinal cord lesions [34]. The updates also introduce biomarkers such as kappa-free light chains and serum neurofilament light chains to aid diagnosis, offering new tools for early MS detection and treatment [33].

MRI remains the gold standard for detecting acute inflammation and distinguishing MS from other neurological conditions [35]. It is recommended that MRI be performed at low field strength (<3T) during pregnancy to minimize fetal exposure, and gadolinium contrast should be avoided due to potential fetal brain toxicity [36,37]. This is especially important postpartum when breastfeeding, although MRI re-scans are recommended two to three months after delivery [38].

The safety of DMTs during pregnancy and breastfeeding is still an area of active research. Preconception counseling is essential to address factors such as maternal disease activity, risks of therapy withdrawal, and postpartum relapse, all of which require individualized care [39]. It is reassuring that most pregnancies in women with MS result in healthy babies, although the highest relapse rates occur postpartum [40]. Long-term disability is linked to relapses in the year prior to conception, underscoring the importance of disease stabilization before pregnancy [41]. An independent Trust review of our care and diagnosis confirmed our diagnosis of MS, despite the patient having only one attack and no contrast enhancement on a postpartum scan with gadolinium. The multidisciplinary review and feedback aligned with the diagnosis of MS.

The patient’s negative anti-aquaporin and anti-MOG antibody tests, along with the presence of oligoclonal bands in the CSF and moderate elevation of white blood cells, further supported the MS diagnosis. Although the patient initially presented with TM and technically had CIS, the MRI showed lesions in both the brain and spinal cord, and the CSF findings met the McDonald criteria for MS. The brain MRI revealed only two lesions, while the spinal cord MRI showed multiple lesions consistent with spinal MS. MRI scans demonstrated intracranial abnormalities in the right centrum semiovale, left parieto-occipital subcortical white matter, and cervical and thoracic cord lesions at C2/C3, C4/C6, and T2/T4, with additional lesions at T11 and T12. These lesions corresponded to the patient’s clinical symptoms, including upper and lower limb weakness, numbness, and bladder dysfunction. Notably, the lesions at C2 and C4 were associated with the patient’s upper limb symptoms, while those at T3, T11, and T12 correlated with her lower extremity dysfunction. The right centrum semiovale lesion could present as hemiparesis or cognitive issues, although it was asymptomatic in this case. Collectively, these MRI findings strongly support an MS diagnosis, distinguishing it from NMOSD or TM.

The patient was offered treatment with a disease-modifying agent, as she met the McDonald criteria for RRMS, despite the potential overlap with NMOSD. Although oligoclonal bands were measured during the patient’s symptomatic pregnancy, which could also be present in NMOSD, the overall clinical and imaging findings favored MS.

This case underscores the unique clinical challenges of diagnosing and managing MS during pregnancy, a rare occurrence given the usual pattern of MS onset. Pregnancy can reduce relapse rates, particularly in the third trimester, but relapse rates often increase postpartum. This report highlights the importance of a multidisciplinary approach and individualized care to minimize risks to both mother and child, emphasizing the need for continued research into MS during pregnancy and better management strategies to improve patient outcomes.

## Conclusions

The relationship between MS and pregnancy is complex, and much remains to be learned about the disease. MS therapy and management during pregnancy are continually evolving, making it essential to stay updated. For this reason, we highlight our unique case presentation. When treating pregnant patients with MS, a multidisciplinary approach is crucial, with a focus on critically analyzing the risks and benefits of various treatment options at different stages of pregnancy. The primary goal is to reduce the risk of relapse during pregnancy and minimize long-term disability and disease progression after childbirth. This case also illustrates the diagnostic challenges of differentiating MS from other neurological conditions during pregnancy. While pregnancy is generally protective against MS relapses, as seen in most patients, this case demonstrates that new-onset MS can still occur, requiring individualized assessment.

The patient’s symptoms, including sensory deficits and paraplegia, combined with MRI findings of spinal cord and brain lesions and positive CSF oligoclonal bands, fulfilled the McDonald criteria for MS diagnosis. These findings highlight the importance of thorough diagnostic evaluation to distinguish MS from other conditions, such as TM and NMOSD. The patient’s successful response to early immunotherapy and plasmapheresis further emphasizes the importance of prompt, individualized treatment in reducing acute disability and improving functional outcomes. At six weeks postpartum, the patient showed significant recovery, including improved mobility and normal sphincter function, underscoring the value of early intervention. This case reinforces the need for a multidisciplinary approach, involving neurology, obstetrics, anesthesiology, and pediatrics, to manage MS during pregnancy and minimize risks to both mother and child. It also highlights the ongoing need for research into the impact of pregnancy on MS onset and progression, particularly in rare cases such as de novo MS during pregnancy.
